# Mumps transmission in social networks: a cohort study

**DOI:** 10.1186/s12879-016-2135-5

**Published:** 2017-01-10

**Authors:** Susan Hahné, Tessa Schurink, Jacco Wallinga, Jeroen Kerkhof, Marianne van der Sande, Rob van Binnendijk, Hester de Melker

**Affiliations:** Centre for Infectious Disease Control, National Institute for Public Health and the Environment (RIVM), PO Box 1, 3720 BA Bilthoven, The Netherlands

**Keywords:** Mumps, Mumps virus, Vaccination, Cohort study

## Abstract

**Background:**

Mumps emerged among highly vaccinated populations in the Netherlands. This offered a unique opportunity to study mumps virus transmission. In particular the extent to which asymptomatic infections in vaccinated people contribute to ongoing mumps virus transmission is uncertain. Insight into this could help project the future burden of mumps in vaccinated populations. We therefore studied the relative infectiousness of symptomatic and asymptomatic cases.

**Methods:**

In a cohort study we followed contacts of notified mumps cases (ring 1) and contacts’ contacts (ring 2) for 40 days to ascertain symptoms of mumps and social contacts by weekly diaries and questionnaires, and mumps virus infections by taking finger stick dried blood spot specimens (DBS) that were tested for mumps-specific IgG antibodies. Mumps IgG concentrations >1500 RU/ml in a single sample, a four-fold increase in IgG antibody concentration in paired samples, or a positive oral fluid PCR were defined as recent infection.

**Results:**

We recruited 99 contacts (40 in ring 1 and 59 in ring 2) of 10 mumps index cases. The median age of participants was 23 years (range 18–57 years), 31 (31%) were male. At study entry, DBS of 4 out of 78 (5%) participants with samples showed serological evidence of recent mumps virus infection. Three of these reported mumps symptoms. Among the 59 participants who provided DBS at the beginning and end of the follow-up period, none had serological evidence of infection during this period. Of 72 participants who provided at least one oral fluid sample, one participant (1%) who also reported mumps symptoms, was found PCR positive. Of all 99 participants, the attack rate of self-reported mumps was 4% (95% CI 1.1–10.0%). Of the 5 laboratory confirmed mumps cases, 1 reported no mumps symptoms (percentage asymptomatic 20% (95% CI 0–71%)). Compared to non-students, students had larger households and more household members who were born after 1980 (*p* < 0.01 and <0.01, respectively).

**Conclusions:**

We demonstrated that this prospective cohort study design allows for inference of the proportion of asymptomatic mumps infections. Because we only detected one asymptomatic mumps virus infection, we could not assess the relative infectiousness of asymptomatic mumps. Household characteristics of students differed from non-students. This may partly explain recent mumps epidemiology in the Netherlands.

## Background

Mumps virus infection can result in symptomatic or asymptomatic infection. Symptoms of mumps include fever, swelling and tenderness of salivary glands, usually the parotid gland [1]. Mumps virus infection can result in complications such as orchitis, meningitis, pancreatitis, and deafness. These complications can also occur in vaccinated individuals. Importantly, the risk of mumps complications is lower among vaccinated compared to unvaccinated cases of mumps [2–4]. A recent study in the Netherlands among a student population with very high mumps vaccine coverage found that two-thirds of individuals with recent mumps virus infection did not report mumps specific symptoms [5]. Mumps virus is transmitted through respiratory droplets. The median incubation period is 19 days (range 15–24 days), with a serial interval of around 20 days [6]. Mumps virus can be isolated from 7 days before to 9 days after onset of symptoms [1]. Vaccination for mumps was introduced in many industrialized countries including the Netherlands during the 1980s. This resulted in a fast decline in the incidence of the disease [1]. However, since the beginning of the 21st century the incidence of mumps has increased in many industrialised countries, including the Netherlands [3]. Mumps has been a notifiable disease in the Netherlands since 1976, except for the period 1999 to 2008. During the latter period, surveillance of mumps was based on laboratory surveillance and the reporting of outbreaks in institutions or schools.

The increase in The Netherlands started in 2004, when a mumps outbreak was reported among students of an international school [7]. Between 2007 and 2009, increased mumps circulation was observed among religious groups with low vaccination coverage [8]. Between 2009 and 2012, a countrywide epidemic occurred mainly among university students and their contacts [3]. This epidemic had clear seasonality, with peaks in spring and autumn. Between September 2009 and August 2012, 1557 cases were notified, two-thirds of whom were aged 18 to 25 years. The majority of cases (68%) occurred in twice MMR vaccinated individuals. The most frequently reported complication was orchitis. Vaccinated cases had a nearly 75% reduced risk of this complication compared to unvaccinated cases. An outbreak investigation suggested that attending a student party, being unvaccinated and living with many other students were important risk factors [9].

A national mumps outbreak management meeting in January 2010 considering the outbreak among vaccinated students concluded there was insufficient evidence to recommend a booster dose of mumps vaccination for the at risk population. Key research priorities identified were: (1) the consequences of mumps virus infection for fertility in male patients with orchitis; (2) the knowledge, attitude and practice of students regarding mumps and mumps vaccination; and (3) the role of asymptomatically mumps virus infected individuals in the dynamics of the epidemic. In this paper we report a study that addressed the third question. Estimating key parameters regarding the proportion of cases that are asymptomatic and their role in spreading mumps virus is needed to understand the outbreak patterns observed to inform projections regarding the future incidence of mumps in The Netherlands and elsewhere. We aimed to study the proportion of mumps virus infections that are asymptomatic and their infectiousness by following cohorts of individuals who are part of a social network around index cases with mumps and therefore at a high risk of mumps infection.

## Methods

Mumps is a notifiable disease in the Netherlands. Notification criteria for mumps include >1 related symptom (i.e., acute onset of painful swelling of the parotid or other salivary glands, orchitis, or meningitis) and laboratory confirmation of infection or an epidemiologic link to a laboratory-confirmed case. We studied mumps virus transmission by a case-contact design, in which contacts of notified mumps cases (ring 1) and their contacts (ring 2) were prospectively followed for 40 days from the date of the start of the study (i.e. the date the first sample was taken) to ascertain symptoms of mumps and contacts by weekly diaries and questionnaires. Mumps was described in the questionnaire as ‘an acute painful swelling of one or both cheeks due to an inflammation of one or more of the salivary glands’). Mumps virus infections were ascertained by sampling specimens for laboratory confirmation of mumps virus infection. The study set up allows for inferring the proportion of mumps infections in ring 1 and 2 that remain asymptomatic. If the number of both symptomatic and asymptomatic infections in ring 1 is sufficiently high, it would allow for inferring the relative infectiousness of asymptomatic cases by contrasting the infection attack rates among contacts of asymptomatic and symptomatic cases, respectively.

Mumps index cases were recruited by Municipal Health Services (MHS), who receive notifications of mumps from clinicians and laboratories. Index cases of mumps with an active social network were asked by the MHS whether they wanted to provide name and contact details of a maximum of 10 contacts who could be invited to participate in the study (respondent driven sampling); these individuals were considered the first ring around an index mumps patient. There were no requirements in terms of having had actual contact. Upon consenting, the contacts in ring 1 were asked to recruit an additional maximum of 10 individuals by spreading a flyer with information about the study (‘ring 2’). This way we established networks of people around a mumps case, in which mumps virus transmission could occur. Index cases were not included as participants. Contacts (ring 1 and ring 2) were eligible for participation if they were ≥16 years of age and did not report mumps in the past. We aimed to include the first ring contacts within 10 days of the first symptoms of mumps disease of the index case.

### Study procedures

Upon entering into the study, participants in ring 1 and 2 were asked to fill in an online questionnaire and an informed consent form, and to submit a dried blood spot specimen (DBS) for mumps specific serology. The DBS was finger stick blood collected by self-sampling by the participants. The questionnaire collected information on vaccination status, composition of the household, history of mumps, education and membership of a (student) association. Participants were further asked to take oral fluid samples for 21 consecutive days upon entry in the study, to allow detection of mumps viral RNA by RT-PCR. In addition, participants were asked to complete a weekly on-line contact-diary, for a period of 3 weeks, starting from the moment of inclusion into the study. This diary collected the following information: whether or not there had been contact with any of the participants in ring 1 and 2 of the index case in the preceding week (a list with all the names was provided); whether there was contact with a person ill with mumps that week; attendance at social events with ≥ 5 participants and if yes, in what setting; and whether there was occurrence of disease symptoms that week (parotitis, orchitis, respiratory infection, fever).

At the end of the follow-up period (day 40 counted from the day of the first sample), participants were asked to submit a second and last DBS and to fill in a second online questionnaire on the occurrence of mumps symptoms in the previous month. When a mobile telephone number was provided, participants were reminded by text messaging to their mobile phone of the study procedures. Upon completion of the study (defined as having filled in the questionnaires, two DBS, and at least 75% of the oral fluid and diary days), participants received a gift voucher of €20. During the study, this incentive was increased to €50.

### Sample size calculation

Assuming a 10% attack rate (AR) among contacts of asymptomatic cases and 50% among contacts of symptomatic cases, we aimed to recruit 20 index cases, who would provide 120 primary contacts (assuming 60% participation rate; i.e. 20 x 6 contacts) and 480 unique secondary contacts (assuming 60% participation rate and taking 33% overlap among primary and secondary contacts into account). Thus, per index case we aimed for 30 contacts (6 in ring 1 and 24 in ring 2).

### Analyses

We calculated the AR of self-reported mumps in the entire study population and among participants who reported to have been in contact with a mumps case. Contact with a mumps case was defined as being a participant in ring 1, reporting to have been in contact with a mumps case, and being a contact of cases identified during the follow-up period. Among participants, we compared social contact patterns of students with those of non-students.

### Microbiological testing

IgG antibody concentrations for mumps in a DBS were tested with fluorescent bead-based multiplex immunoassay (MIA) using Luminex technology [10]. Antibody concentrations were expressed in RIVM units per milliliter (RU/ml), previously standardized [5]. In oral fluid samples, the presence of mumps virus RNA (F-gene) was detected by using a realtime Taqman PCR as previously described [11]. Recent mumps virus infection was defined as a positive PCR for at least one oral fluid sample, a fourfold increase in mumps specific IgG antibody concentration in paired DBS and/or a mumps specific IgG antibody concentration ≥1500 RU/ml in a single serum sample [5].

The study was approved by the medical ethical committee ‘Noord Holland’ (http://www.metc.nl/), protocol number M011-044.

## Results

Between mid-November 2011 and mid-April 2012, 55 index cases of mumps notified to municipal health services (18% of all notified cases (*n* = 305) in this period) were asked to invite contacts to participate in the study. Of these 55, a contact network could be established for 10 index cases (18%). Nine of the index cases were laboratory confirmed, one was notified based on a clinical picture of mumps and an epidemiological link to a laboratory confirmed case. The 10 networks were situated in five student cities/locations: Nijmegen (3), Groningen (3), Amersfoort (1), Maastricht (1), Nyenrode (1) and Wageningen (1) (Table 1). These networks included 40 participants in ring 1 (range of the number of participants in ring by index case: 1–8 persons) and 59 participants in ring 2 (range: 2–11 persons). None of the participants was present in both ring 1 and 2. The total number of participants was 99. The median age of participants was 23 years (range 18–57 years), 31 (31%) were male. Of 95 participants of whom information was known, 79 (83%) indicated that they participated in the national immunisation programme. Seventy-four (78%) were in higher education (full-time), of whom 52 (55% of total) were university students.

**Table 1:** Timing of study procedures by network

Location of network	Date of onset (DOO) index	Median (range) number of days between DOO index and start sampling among contacts in ring 1^b^	Median (range) number of days between DOO index and start sampling among contacts in ring 2
1. Nijmegen	21-11-2011	30 (24–42)	45 (43–50)
2. Groningen	1-1-2012	19 (17–29)	30 (19–33)
3. Groningen	19-1-2012	26 (21–30)	32 (23–41)
4. Nijmegen	21-1-2012	23 (23–25)	31 (23–56)
5. Groningen	13-3-2012	20 (14–24)	30 (22–60)
6. Amersfoort	29-2-2012	28 (28–54)	34 (30–37)
7. Maastricht^a^	30-3-2012/20-3-2012	57 (57–57)	69 (69–72)
8. Nyenrode	25-3-2012	38 (38–38)	36 (27–40)
9. Wageningen	29-3-2012	27 (27–27)	36 (33–52)
10. Nijmegen	27-3-2012	31 (24–37)	48 (35–48)

^a^There were two index cases in this cluster, the first date of onset is used to compute the median days between DOO index and the start of sampling

^b^Median of these medians is 28 days

### Results at study entry

Of the 99 participants, 78 provided a DBS at the start of their sampling period. Of these 78, four (5%) had an IgG concentration above 1500 RU/ml, indicative of recent mumps virus infection. Of these, three reported to have had mumps in 2012, of which one with a date of onset 2 days prior to the index case and two with a date of onset after the index case (4 and 25 days, respectively). Considering these onset dates, these cases were classified as co-primary (*n* = 2) and secondary (*n* = 1). The remaining individual with serologic evidence of recent infection at study entry reported not to have had symptoms of mumps in the past. One of the 99 participants reported to have had mumps before 2012 (in 1967). He had an IgG concentration below the threshold for recent infection. Of the 21 participants who did not submit a DBS at entry into the study, none reported having had mumps in the past.

### Follow-up

Of the 99 participants, 60 (61%) sent in oral fluid samples for at least 16 days. Of 72 participants who provided at least one oral fluid sample, one (1.4%) was found PCR positive for mumps virus. This participant also reported to have had mumps symptoms in the first week of follow-up. Her DBS at study entry had no serological evidence of recent mumps virus infection. She did not submit a DBS at the end of the study. Among 59 participants who submitted a DBS at the beginning and the end of the follow-up period, none had evidence of seroconversion. In total five cases of mumps virus infection were found (4 based on a high titer in the DBS at entry into the study and one PCR positive case during follow-up). Of these, one (20%) did not report mumps symptoms (proportion asymptomatic 20% (95% CI 0–71%). Since there was only one asymptomatic infection, we could not reliably study infectiousness of asymptomatic mumps virus infected individuals.

The AR of self-reported mumps among participants was 4% ((4/99) (95% confidence interval (CI) 1.1–10.0%). The AR among participants who reported to be exposed to mumps was 7% ((1/15), 95% CI 0.2–32%).

### Social contact patterns

Of all 99 participants, 96% (95% CI 93–98%) reported having attended a gathering of five people or more during the past week (average proportion over 3 weeks); 35% (95% CI 29–42%) attended at least one party; and 17% (95 CI 13–23%) attended a dancing. The median number of household members (excluding the participant) was 3 (range 0–9), of whom a median 2 (range 0–9) was born after 1980. Compared to non-students, students had larger households and more household members who were born after 1980 (*p* < 0.01 and <0.01, respectively) (Table 2). Of the 89 participants who filled in at least one of the weekly contact diaries, 15 (17%) reported to have had direct contact with someone with mumps. One of these 15 reported mumps symptoms during the follow-up period.

**Table 2:** Household size and age of participants by student status

Household characteristics	University students (*n* = 52)	Non-students^a^ (*n* = 18)	*p*-value
Median household size, excluding participant (range)	4.0 (0.0–9.0)	1.0 (0.0–4.0)	<0.01
Median number of household members born after 1980, excluding participant (range)	3.0 (0.0–9.0)	0.5 (0.0–4.0)	<0.01

^a^Students other than full-time university students (e.g. part-time students or non-university students) are excluded here

## Discussion

In response to the emergence of mumps in a highly vaccinated adolescent population in the Netherlands, we set out to document key transmission parameters in social networks around mumps cases, using a respondent driven design. The key finding of our study is that the attack rate of symptomatic mumps was 4% among participants. This is higher than the 2% symptomatic mumps AR found in a large serological study, especially when considering the much longer follow-up period of the latter study (average 26 months) [5]. The AR we found is, however, much lower than the ARs of 13% and 22% found in outbreaks of mumps affecting adolescent populations with a similar vaccination coverage [9, 12]. The median age of participants to our study (23 years) matched the age of cases of mumps during the epidemic, with the majority being 18–25 years [3]. This is therefore not an explanation for the difference in ARs. The key characteristic of the outbreaks with a high AR was that they occurred among attendees of a party [9, 12]. This suggests particular circumstances favouring transmission occurred during these parties that were not present in our networks around a mumps case, even though a relatively large proportion of our participants did attended social gatherings and parties. A study from the US suggested that a high infectious dose is needed to overcome vaccine induced immunity [13]. This intense exposure was apparently not very frequent among our participants.

Four of our participants (5%) had evidence of recent infection upon study entry, based on high IgG titres in the DBS taken at entry into the study. The main explanation for this relatively high proportion is that sampling in our study started at a median of 28 days after onset of symptoms in the index case (Table 2), which is considerably later than the 10 days we aimed for. This delay was caused by difficulties to recruit participants. In future studies, exploring how to include participants faster is recommended but will be challenging using a respondent driven design.

Since we detected only one asymptomatic mumps virus infected individual, we were not able to study the relative infectiousness of this compared to symptomatic cases. This is related to the low number of participants we managed to include. Even despite increasing the incentive, it was difficult to recruit a sufficient number of participants. This may be related to the quite demanding nature of study procedures, including self-sampling of DBS, oral fluid and filling out questionnaires. However, to detect and assess infectiousness of asymptomatic mumps virus infections, the intense microbiological sampling as conducted in our study is a prerequisite.

We observed that students among our participants had more intense social behaviour than non-students in our study. Their households consisted more often of young people (born after 1980). Both factors are consistent with the observation that the mumps outbreak in 2009–2012 mostly affected students [3, 5].

## Conclusions

Our prospective cohort study design with extensive laboratory testing of participants allowed for inference of the proportion of asymptomatic mumps infections. The AR in our cohort was somewhat higher than the AR found in another prospective follow-up study, but lower than in party related outbreaks in the Netherlands. Because we only detected one asymptomatic mumps virus infection, we could not study the relative infectiousness of asymptomatic mumps. Household characteristics of students differed from non-students. This may partly explain recent mumps epidemiology in the Netherlands. Recent surveillance data suggest that since 2012 circulation of mumps among students has declined, most likely since levels of immunity in this population have increased as a result of the outbreak [3, 14]. It is likely that once a sufficient number of susceptible new students has accrued, mumps will re-emerge. Since serological correlates for protection for mumps are uncertain, sero-epidemiological studies are of limited use to assess this risk [5, 15]. Mathematical models may be able to project when this re-emergence is likely to occur [16]. This is important so that control measures and outbreak studies can be prepared.

## Abbreviations

AR: attack rate
DBS: dried blood spot specimens
IgG: Immunoglobulin G
MMR: measles, mumps, rubella vaccine
PCR: polymerase chain reaction
